# The transcriptome of corona radiata cells from individual MІІ oocytes that after ICSI developed to embryos selected for transfer: PCOS women compared to healthy women

**DOI:** 10.1186/s13048-014-0110-6

**Published:** 2014-11-29

**Authors:** Marie Louise Wissing, Si Brask Sonne, David Westergaard, Kho do Nguyen, Kirstine Belling, Thomas Høst, Anne Lis Mikkelsen

**Affiliations:** Department of Gynecology-Obstetrics, Holbaek Fertility Clinic, Holbaek Hospital, Smedelundsgade 60, 4300 Holbaek, Denmark; Institute of Biology, University of Copenhagen, 2100 Copenhagen, Denmark; Department of Systems Biology, Center for Biological Sequence Analysis, Technical University of Denmark, Kemitorvet building 208, 2800 Lyngby, Denmark; DTU Multi Assay Core, Technical University of Denmark DTU, 2800 Lyngby, Denmark

**Keywords:** Corona radiata cells, Transcriptome, Gene expression, PCOS, Oocyte quality

## Abstract

**Background:**

Corona radiata cells (CRCs) refer to the fraction of cumulus cells just adjacent to the oocyte. The CRCs are closely connected to the oocyte throughout maturation and their gene expression profiles might reflect oocyte quality. Polycystic ovary syndrome (PCOS) is a common cause of infertility. It is controversial whether PCOS associate with diminished oocyte quality. The purpose of this study was to compare individual human CRC samples between PCOS patients and controls.

**Methods:**

All patients were stimulated by the long gonadotropin-releasing hormone (GnRH) agonist protocol. The CRC samples originated from individual oocytes developing into embryos selected for transfer. CRCs were isolated in a two-step denudation procedure, separating outer cumulus cells from the inner CRCs. Extracted RNA was amplified and transcriptome profiling was performed with Human Agilent® arrays.

**Results:**

The transcriptomes of CRCs showed no individual genes with significant differential expression between PCOS and controls, but gene set enrichment analysis identified several cell cycle- and DNA replication pathways overexpressed in PCOS CRCs (FDR < 0.05). Five of the genes contributing to the up-regulated cell cycle pathways in the PCOS CRCs were selected for qRT-PCR validation in ten PCOS and ten control CRC samples. qRT-PCR confirmed significant up-regulation in PCOS CRCs of cell cycle progression genes *HIST1H4C* (FC = 2.7), *UBE2C* (FC = 2.6) and cell cycle related transcription factor *E2F4* (FC = 2.5).

**Conclusion:**

The overexpression of cell cycle-related genes and cell cycle pathways in PCOS CRCs could indicate a disturbed or delayed final maturation and differentiation of the CRCs in response to the human chorionic gonadotropin (hCG) surge. However, this had no effect on the *in vitro* development of the corresponding embryos. Future studies are needed to clarify whether the up-regulated cell cycle pathways in PCOS CRCs have any clinical implications.

**Electronic supplementary material:**

The online version of this article (doi:10.1186/s13048-014-0110-6) contains supplementary material, which is available to authorized users.

## Background

Polycystic Ovary Syndrome (PCOS) is the most prevalent endocrine disorder of women in the reproductive age and represents a combination of polycystic ovaries, oligo/anovulation and hyperandrogenism [1]. The follicular microenvironment previously found to be altered in PCOS women might influence oocyte maturation and oocyte developmental competence [2,3]. Previous studies of PCOS women compared to healthy women revealed gene expression differences in metaphase ІІ (MII) oocytes [4], in cumulus cells of individual MІІ oocytes with unknown developmental potential [5] and pooled, cultured cumulus cells [6]. Ribosomal RNA content was increased in cumulus cells of PCOS women [7], which could indicate a higher rate of proliferation; also granulosa cells from PCOS women have been shown to be hyperproliferative [3]. These alterations may suggest an altered oocyte quality in PCOS patients compared to controls. However, they do not necessarily extrapolate to the clinical situation, where only oocytes developing into top quality embryos are used for transfer. A meta-analysis showed that PCOS women had a similar number of top quality embryos and similar rates of pregnancy and live births compared to healthy women undergoing *in vitro* fertilization (IVF), but PCOS patients had more oocytes retrieved and a significantly lower fertilization rate [8].

Corona radiata cells (CRCs) refer to the innermost layer of the cumulus cells, which is in direct contact with the zona pellucida of the oocyte. Throughout folliculogenesis and until the luteinizing hormone (LH) surge for final oocyte maturation, transzonal projections exist between the oocyte and the CRCs, allowing exchange of substances between the oocyte and the CRCs [9]. We hypothesize that transcriptomic analysis of CRCs would serve as a non-invasive method of gaining deeper understanding of the microenvironment of the oocyte. Since PCOS and non-PCOS women undergoing IVF had the same clinical outcome [8], we wanted to find out whether the transcriptomic profile of CRCs would differ between PCOS and controls in clinically relevant samples of CRCs from embryos chosen for transfer.

## Materials and methods

This study was approved by The Danish Ethical Science Committee (SJ-156) and conducted in accordance with the Helsinki Declaration and all participants gave informed consent before inclusion in the study.

### Study population

Ten women with PCOS and ten healthy, regularly cycling women without known disease (controls) were included. Exclusion criteria were diabetes type 1 or 2, impaired thyroid, renal or hepatic function, congenital adrenal hyperplasia, endometriosis, premature ovarian failure, hypothalamic amenorrhea or age >35 years.

Diagnosis of PCOS was made according to the Rotterdam Consensus Criteria [1]. For all control women, indication for intracytoplasmic sperm injection (ICSI) was a partner with infertility (defined as <5 million progressively moving spermatozoa/ml). For the PCOS women, half (5/10) of the couples were referred to ICSI because of male infertility and the rest after 4–6 failed attempts of intra-uterine insemination (IUI).

#### Baseline examination

Participants were included in the study based on a focused gynecological history and objective examination including transvaginal ultrasound of the ovaries and uterus. Blood samples were drawn after an overnight fast at 08.30-09.00 a.m. on cycle day (cd) 3–5 for regularly cycling women and on a random day for amenorrhoeic women. All androgen analyses were done at the same laboratory (Statens Serum Institut, SSI, Copenhagen, Denmark) in order to minimize variability. Total testosterone was measured by the CHS™ MSMS Steroids Kit (PerkinElmers®, Waltham, Massachusetts, USA) with intra-assay variation of 9.6% and inter-assay variation of 10.6%. Sex hormone-binding globulin (SHBG) was measured by Architect i2000 analyzer (Abbott®, Abbott Park, Illinois, USA) with an intra-assay variation of 2.8% and an inter-assay variation of 5.8%. Free testosterone was calculated from total testosterone and SHBG [10]. LH and follicle-stimulating hormone (FSH) were measured by immunoassay (LH: ref 11732234, FSH: ref 11775863, Roche Diagnostics, Mannheim, Germany).

#### Ovarian stimulation

Ovarian stimulation was achieved by the long gonadotropin-releasing hormone (GnRH) agonist protocol. Pituitary desensitization with buserelin 0.5 mg (Suprefact®, Sanofi-Aventis, Paris, France) was started on cd 21 in regularly menstruating women and at cd 15 for oligo/amenorrhoeic women starting with ethinylestradiol 30 mg/desogestrel 150 mg daily from the 1^st^ day of bleeding (Marvelon®, Organon/Microgyn®, Bayer Pharma, Leverkusen, Germany) and until cd 21. Controlled ovarian stimulation with recombinant FSH (rFSH) (Puregon®, Organon, Oss, Netherlands) was started after at least 14 days of desensitization. Follicle growth was monitored by transvaginal ultrasound. Recombinant human chorionic gonadotropin (rhCG) 6500 IU (Ovitrelle®, Modugno, Italy) was administered when at least three follicles reached the size of 17 mm. Oocyte Pick-up (OPU) was performed 36 hours later under transvaginal ultrasound guidance. Luteal phase support (Lutinus®, Ferring©, Copenhagen, Denmark) was given from the day of transfer and until the pregnancy test. We adhered to the Danish National Criteria of elective single transfer for all patients <37 years of age. Transvaginal ultrasound was performed three weeks after a positive hCG blood test to confirm intrauterine clinical pregnancy.

### Isolation of corona radiata cells, fertilization and time lapse incubation of embryos

Following OPU, cumulus-oocyte complexes were washed several times in Fertilization medium (Cook, Eight Mile Plains, Queensland, Australia) to remove cell debris, and incubated for two hours in Fertilization medium (Cook, Eight Mile Plains, Queensland, Australia). Then the oocytes were transferred to a droplet of Cleavage medium (Cook, Eight Mile Plains, Queensland, Australia) and denudated in a two-step procedure: First, the cumulus cells were removed by gentle pipetting in 20 μl Cumulase® (Origio, Måløv, Denmark) with a 1–10 μl Eppendorf Pipette using a Dual filter PCR clean 20 μl tip (Eppendorf, Hamburg, Germany). Then the oocyte with the remaining CRCs was transferred to 10 μl Cumulase® (Origio, Måløv, Denmark) and the CRCs were removed by gentle pipetting with a Denudation pipette 0.134-0.145 mm (Vitrolife, Göteborg, Sweden). Immediately after oocyte denudation, individual droplets containing the CRCs were transferred to DNA Lobind Eppendorf tubes (Eppendorf, Hamburg, Germany), snap frozen in liquid nitrogen and stored at −80°C until RNA extraction. MΙΙ oocytes were fertilized by ICSI within ten minutes after denudation, and incubated in individual wells in an Embryoscope® (Unisense Fertilitech, Aarhus, Denmark), which gave the opportunity to track the development of all fertilized oocytes until transfer (day 2), or, for the untransferred embryos, until vitrification as top quality blastocyst or disposal at day 5/6. Analysis of the images was done with the EmbryoViewer® Software (Unisense FertiliTech Aarhus, Denmark). The time from ICSI to the following events were annotated: Pronuclei breakdown (defined as the first picture frame where the pronuclei disappeared), 1^st^ cleavage (defined as the first picture frame where the zygote turned into two cells) and cleavage to four cells (defined as the first picture frame where four cells were observed the first time).

### Selection of CRC samples used in the study

The CRC samples used in this study came from oocytes developing into top quality embryos: All transferred embryos were top quality embryos according to the ALPHA/ESHRE consensus [11] scoring points in short: Four cells at day 2, low fragmentation (cut-off 25% fragmentation), cell cycle specific cell size, no multinucleation. Vitrified top quality blastocysts day 5/6 had score 3–6 AA/AB according to the blastocyst classification proposed by Gardner and Schoolcraft [12]. The fate of the ten oocytes corresponding to the CRC samples used were as follows: In the PCOS group, ten transferred embryos gave three clinical pregnancies, resulting in two live births and one missed abortion in gestational week 7 and seven negative hCG tests 14 days after oocyte retrieval; In the control group, three top quality blastocysts were vitrified for later use, and seven transferred embryos gave three clinical pregnancies with two live births and one missed abortion in gestational week 7.

### RNA extraction and amplification

Total RNA was extracted by the RNAequeous® micro-kit from Ambion (Life Technologies, Paisley, UK) according to manufacturer’s instructions. The samples were analyzed for total RNA concentration by Qubit® (Life Technologies, Paisley, UK) and total RNA quality and level of degradation using an Agilent 2100 Bioanalyzer and RNA 6000 Pico LabChip according to the manufacturer’s instructions (Agilent Technologies, Waldbronn, Germany). All of the RNA samples showed two distinct peaks representing 18S and 28S rRNA, which indicated good quality RNA and presented RNA Integrity Number (RIN) from 6–9.4. Total RNA of 50 ng was amplified and converted into cDNA using the Ovation Pico WTA System V2 RNA Amplification System from NuGEN® Inc. (NuGEN®, San Carlos, California, USA).

### Microarray experiment

cDNA was coupled to a Cyanine 3-dUTP fluorescent dye (Cy3) using the Oligonucleotide Array-Based CGH for Genomic DNA Analysis, Enzymatic Labeling for Blood, Cells or Tissues (protocol version 6.2, Agilent Technologies, Santa Clara, California, USA). Cy3-labeled cDNA was hybridized to Agilent Human Gene Expression Microarrays 4 × 44k v2 (G4845A) using the One-Color Microarray-Based Gene Expression Analysis, Quick Amp Labeling (version 5.7 protocol, Agilent Technologies, Santa Clara, California, USA) and scanned using an Agilent DNA Microarray scanner (Agilent Technologies, Santa Clara, California, USA). For microarray analysis, we used six CRC samples from PCOS women, and six from healthy controls (12 arrays in total).

### Quantitative reverse-transcriptase PCR

The following TaqMan® Gene Expression Assays (pre-designed) (Applied Biosystems, Life Technologies Europe, Nærum, Denmark) were used (Assay ID-No: Hs00168719_m1 (Cyclophillin B/PPIB), Hs00171034_m1 (Cyclin T2, CCNT2), Hs00543883_s1 (histone cluster 1, H4c/HIST1H4C), Hs00608098_m1 (E2F transcription factor 4, p107/p130-binding/E2F4), and Hs00964100_g1 (ubiquitin-conjugating enzyme E2C/UBE2C)). Sample triplicates were prepared according to the manufacturer’s instructions. A total reaction volume of 20 μL was prepared on ice containing 10 μL TaqMan® Gene Expression Master Mix (2X), 1 μL TaqMan® Gene Expression Assay Mix (20X), 4 μL cDNA 5 ng/μl, and 5 μL RNase-free water. The samples were then centrifuged at 1,100 × g at 4°C for 5 minutes.

Gene expression was quantified using the MX3005 qPCR system (Agilent Technologies Denmark, Hørsholm, Denmark) under the following thermal cycling conditions: 95°C for 10 minutes, 40 cycles of 95°C for 15 seconds and 60°C for 1 minute. The data was normalized to *cyclophillin* (PPIB) [13], and relative quantification calculated according to the Comparative CT Method (ABI user bulletin # 2, 2001). qRT-PCR was performed on ten PCOS CRC samples and ten control CRC samples.

### Gene expression microarray processing and analysis

Array quality was assessed with the arrayQualityMetrics R package [14], which used a variety of statistical tests combined with data visualization to mark outliers. Evaluation was done manually on a per array basis. Pre-processing of microarray data was done with the LIMMA software [15,16] available from the Bioconductor project [17]. Normalization between arrays was done by quantile normalization [18]. For genes with multiple probes, the intensity was defined as the median of all probes mapping to that gene.

Statistically significant differences between PCOS and control CRC samples in expression of individual transcripts were assessed by the LIMMA moderated t-test [16] using the Benjamin-Hochberg method of correction of P-values for multiple testing.

Statistical significance of biological themes was investigated for the entire dataset with the Gene Set Enrichment Analysis (GSEA) software version 2.0.12 (http://www.broadinstitute.org/gsea/index.jsp) [19,20]. In short, the GSEA algorithm ranked genes according to their expression level. By default, genes were ranked using the Signal-2-Noise metric, a more robust measure than both mean and median values, and very robust against outliers:

$$ Signal2 Noise=\frac{\mu_{pcos}-{\mu}_{control}}{s{d}_{pcos}+s{d}_{control}} $$

The enrichment of a pathway was assessed by walking through the list of ranked genes, incrementing a running sum score when encountering a gene found in the pathway, and decreasing it when encountering a gene not in the pathway. According to the authors, this corresponded to a weighted Kolmogorov-Smirnov-like statistic. Statistical significance was assessed by permutating the ranked list of genes a thousand times. Genes which were either poorly expressed, had high intra-variation in the group or had low variance between the PCOS and control group populated the middle of the ranked list, and thus did not contribute to the running sum score. We specifically investigated the pathways contained in the databases KEGG [21] and REACTOME [22]. Normalized enrichment score (NES) is defined as:

$$ \mathrm{N}\mathrm{E}\mathrm{S}=\frac{\mathrm{actual}\ \mathrm{E}\mathrm{S}}{mean\ \left(\mathrm{E}\mathrm{S}\mathrm{s}\ \mathrm{against}\ \mathrm{all}\ \mathrm{permutations}\ \mathrm{of}\ \mathrm{the}\ \mathrm{dataset}\right)} $$

NES was reported together with the false discovery rate (FDR). We reported pathways with FDR ≤0.05.

All samples were MIAME compliant and were handled according to SOP in the microarray Center. The 12 arrays were submitted to ArrayExpress (http://www.ebi.ac.uk/arrayexpress/) at EMBL using MIAMExpress. The experiment accession number is E-MEXP-3985.

### Statistics

Differences in baseline parameters between groups were tested by Mann–Whitney test (Graphpad Prism v. 6, San Diego, California, USA). Differences between groups in developmental timing of the embryos corresponding to the selected CRC samples were evaluated by Students t-test (Graphpad Prism v. 6, San Diego, California, USA). Differences between PCOS and control CRC samples in gene expression of selected genes in the qRT-PCR experiment were evaluated by Bayesian parameter estimation [23]. A comparison was considered significant if the 95% HDI (Highest Density Interval) did not contain the value 0.

## Results

### Baseline parameters of study groups

The PCOS and control group differed according to PCOS status. The PCOS group exhibited oligomenorrhea, polycystic ovaries and hyperandrogenaemia (Table 1). Age, BMI and basal FSH were similar across groups (Table 1). Embryo kinetic timings of the embryos corresponding to the selected CRC samples did not differ between groups (Table 1).

**Table 1:** **Baseline descriptive parameters of the study participants and the average developmental timings of the embryos corresponding to the CRC samples used in the study**

	**PCOS**	**Controls**	**p-value**
**Age**	27,3±3,4	27,8±4	ns
**BMI**	24±4,8	22,4±3,5	ns
**No of antral follicles/ one plane**	15,8±5,4	8,2±1,7	<0,0001
**No of menstrual bleedings/year**	1,8±3,2	12±0	<0,0001
**Total testosterone**	2,8±1,1	1,2±0,3	0,0003
**Free testosterone**	0,03931±0,02532	0,014±0,0042	0,0029
**SHBG**	78±26	79±21	ns
**LH/FSH**	1,6±0,8	0,8±0,3	0,03
**Time of 2PN breakdown (h)**	23.2±3.8	21.8±2.4	ns
**Time of 1st Cleavage (h)**	26±4.1	24.3±2.5	ns
**Time of cleavage to 4 cells (h)**	38.3±5	36.4±4.2	ns

p-value <0,05 considered significant. Not significant = ns.

### Quality of the arrays

There were no apparent outliers based on the arrayQualityMetrics reports.

### Differentially expressed genes

After correction for multiple testing, no individual genes in the microarray experiment showed significant differential expression between PCOS CRC samples and control CRC samples (Additional file 1).

### Gene set enrichment analysis

GSEA showed upregulation of 24 pathways with FDR < 0.05 in PCOS CRCs compared to control CRCs (Table 2). Especially pathways involved in cell cycle and DNA replication were up-regulated in PCOS CRCs (Table 2). Additional file 2 shows the full pathway list with the genes contributing to the up-regulation of these pathways in PCOS CRC samples.

**Table 2:** **Gene set enrichment analysis of the transcriptome of CRCs of individual oocytes developing into embryos selected for transfer**

**Pathway**	**No of genes enriched/total number of genes in the pathway**	**FDR**	**NES**
**Reactome Cell Cycle**	127/421	0.001	0.42
**Reactome G2 M Checkpoints**	17/45	0.001	0.61
**KEGG DNA Replication**	20/36	0.001	0.65
**Reactome Meiotic Recombination**	35/86	0.001	0.59
**Reactome Packaging of Telomere Ends**	25/48	0.001	0.63
**Reactome Activation of the Pre Replicative Complex**	14/31	0.001	0.60
**Reactome RNA POL Ι RNA POL ΙΙΙ and Mitochondrial Transcription**	45/122	0.001	0.53
**Reactome RNA POL Ι Transcription**	35/89	0.001	0.55
**Reactome RNA POL Ι Promotor Opening**	32/62	0.001	0.64
**Reactome Activation of ATR in Response to Replication Stress**	15/38	0.001	0.63
**Reactome Deposition of new CENPA containing Nucleosomes at the Centromere**	34/64	0.001	0.60
**Reactome DNA strand Elongation**	20/30	0.001	0.69
**Reactome Telomere Maintenance**	39/75	0.001	0.61
**Reactome Cell Cycle Mitotic**	94/325	0.01	0.41
**Reactome Meiosis**	45/116	0.01	0.49
**Reactome Amyloids**	34/83	0.01	0.51
**Reactome lagging strand synthesis**	12/19	0.01	0.69
**Reactome Mitotic M M G1 Phases**	55/137	0.03	0.42
**Reactome Mitotic Prometaphase**	32/87	0.03	0.47
**Reactome DNA replication**	56/192	0.03	0.42
**Reactome Meiotic Synapsis**	34/73	0.03	0.50
**Reactome Chromosome Maintenance**	55/122	0.03	0.42
**Reactome Extension of Telomeres**	13/27	0.03	0.59
**Reactome Transcription**	61/210	0.04	0.41

PCOS women compared controls (n = 6 PCOS arrays vs n = 6 control arrays). Enriched pathways with FDR <0.05 were listed. NES = normalized enrichment score. All enriched pathways were upregulated in PCOS CRC samples.

### Quantitative reverse transcriptase PCR

**Figure 1:**
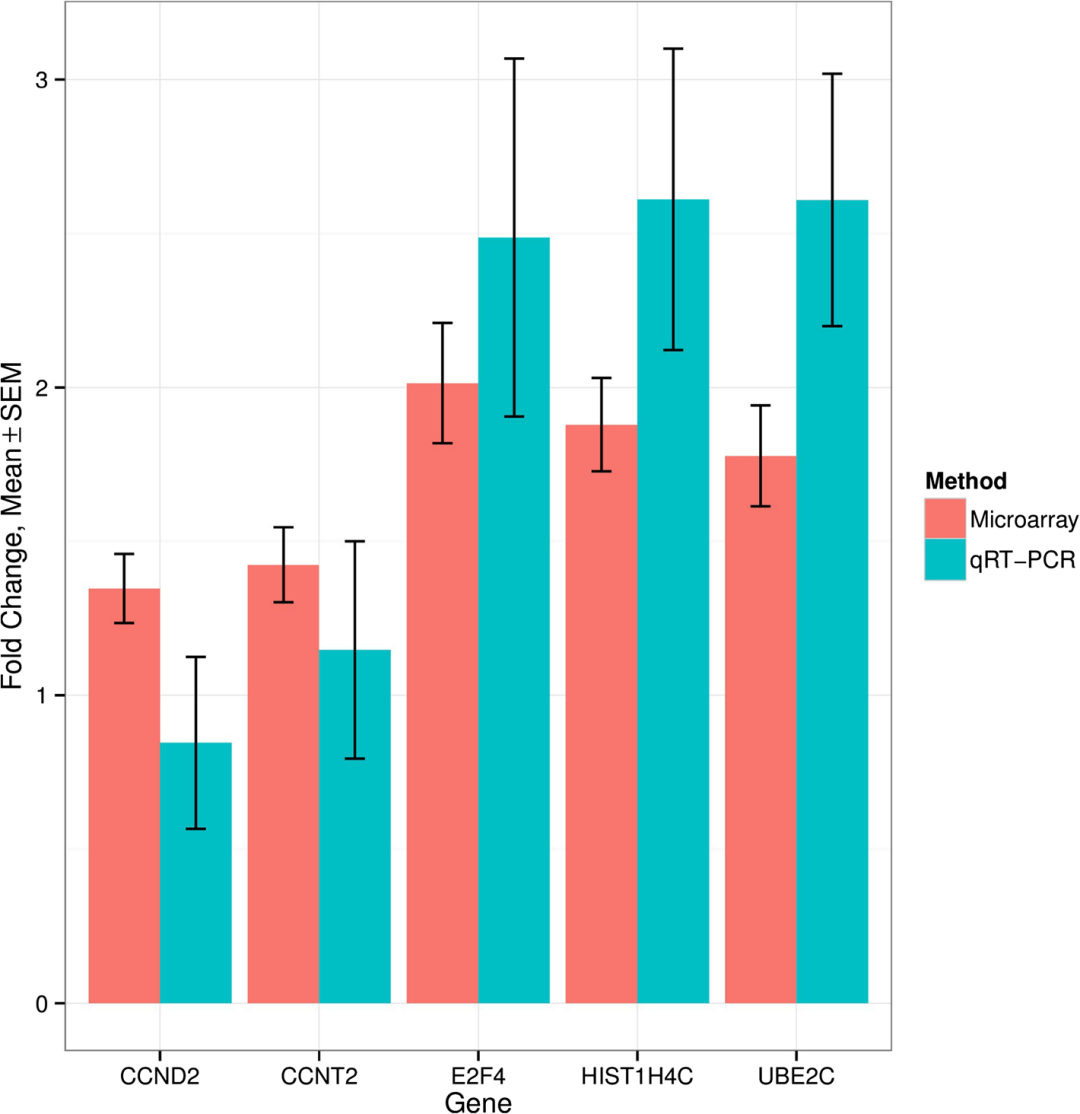
**Comparison between microarray and qRT-PCR results for 5 selected genes in PCOS and control CRC samples.** Fold change (PCOS/controls) ± Standard Error of the Mean (SEM). Red bars represented microarray results and blue bars represented qRT-PCR results. Microarray experiment: 6 PCOS CRC arrays vs. 6 control CRC arrays. qRT-PCR experiment: 10 PCOS CRC samples vs. 10 control CRC samples. The up-regulation of *UBE2C, HIST1H4C* and *E2F4* in PCOS CRCs found in the microarray experiment was in line with the qRT-PCR results, whereas the 1.4 fold up-regulation of *CCND2* and *CCNT2* in PCOS CRCs found in the microarray experiment could not be confirmed by qRT-PCR.

Five of the genes contributing to the up-regulated cell cycle pathways in the PCOS CRCs were selected for qRT-PCR validation. We selected genes with different functions in the cell cycle. Gene expression by qRT-PCR were in accordance with the microarray data for three out of five genes tested (Figure 1). According to the qRT-PCR results, PCOS CRC samples showed significant up-regulation of *HIST1H4C* (FC = 2.7, 95% HDI 0.436-4.49), *UBE2C* (FC (Fold Change) = 2.6, 95% HDI 0.00438-3.21) and *E2F4* (FC = 2.5, 95% HDI 0.571-5.33). *CCND2* and *CCNT2* showed equal expression between groups in the qRT-PCR experiment while the microarray data showed a 1.4 higher expression in the PCOS CRC samples (Figure 1).

## Discussion

In this study, we present for the first time a transcriptomic analysis of individual human CRC samples from oocytes used for transfer or blastocyst vitrification in PCOS and controls. We did not find any significantly differentially expressed individual genes after correction for multiple testing in the present microarray study of PCOS CRCs and control CRCs from MІІ oocytes developing into top quality embryos. This is in contrast to the study by Haouzi *et al.* [5] who found 3,700 significantly differentially expressed genes between PCOS and control cumulus cells from MІІ oocytes of unspecified developmental potential with the same number of arrays per group as in the present study (six PCOS vs. six control arrays). However, the apparent inconsistency between the present study and the study by Haouzi *et al.* [5] could be explained by the different study designs and experimental approaches: Firstly, we used enzymatically isolated CRCs, which constitute the innermost cells closest to the oocyte, whereas Haouzi *et al.* [5] used mechanically cut cumulus cells. Several previous studies have successfully used enzymatically isolated cumulus cells [24–29] and CRCs [30,31] to investigate gene expression differences according to e.g. oocyte quality. Cumulase® used for denudation is a pure, recombinant and specific hyaluronidase and we would not expect the short exposure to mask gene expression differences. To the best of our knowledge, no studies have investigated gene expression differences according to isolation procedure of cumulus cells. Secondly, we used CRC samples from MІІ oocytes capable of developing into top quality embryos, whereas in former studies on gene expression in cumulus cells from PCOS patients [5,6], the developmental potential of the oocytes was not tracked. The strict use of CRCs from MІІ oocytes developing into top quality embryos in the present microarray study might explain why there were no individual genes with significant differential expression between PCOS and controls. Previous studies have shown that the transcriptome of cumulus cells from MІІ oocytes of poor developmental competence differed from cumulus cells of MІІ oocytes with high developmental competence [24,26,29,31–36]. These previous findings underlined the importance of oocyte selection, when comparing cumulus cells or CRCs in relation to a certain condition, such as PCOS.

During the study period, 10% (5/51) of women diagnosed with PCOS did not develop embryos for transfer or blastocyst vitrification. In the present study, we did not examine CRCs from this minority of the PCOS patients. This might have led to an underestimation of the differences in CRC transcriptomic profile between PCOS patients and controls since all controls developed embryos useful for transfer during the study period. Nevertheless, our data were applicable to the majority of the PCOS patients who developed embryos suitable for transfer: Transcriptomic aberrations found in the cumulus cells of MІІ oocytes of unknown developmental potential from PCOS women [5] or in the cumulus cells of the entire oocyte pool from PCOS women [6] did not extrapolate to the transcriptome of CRCs of MІІ oocytes developing into embryos used for transfer or blastocyst vitrification. This indicated that the microenvironment constituted by the CRCs did not differ substantially between PCOS and controls for oocytes with good developmental potential and it is in line with previous findings of similar implantation and pregnancy rates in PCOS patients and non-PCOS patients undergoing IVF [8].

The minority of PCOS patients with no embryos suitable for transfer constituted a clinically interesting subgroup with clearly impaired oocyte quality, and future studies on oocyte quality in PCOS should focus on this subgroup as a distinct entity within the PCOS population.

The GSEA **s**howed up-regulation of pathways involved in cell cycle and DNA replication in PCOS CRCs. This is in line with previous studies showing hyperproliferative cumulus [7] and granulosa [3] cells in PCOS. The LH/hCG surge for final oocyte maturation dramatically down-regulate cell cycle genes in human granulosa cells [37] as well as in rodent cumulus-oocyte-complexes [38]. In agreement with this, cell cycle pathways were up-regulated in human cumulus cells of MІ oocytes compared to cumulus cells of MІІ oocytes [39]. We speculate that the up-regulation of cell cycle pathways in PCOS CRCs could be an indicator of disturbed or delayed final maturation of the cumulus cells/CRCs in response to the LH/hCG trigger.

The three cell cycle-related genes which showed significant up-regulation in PCOS CRCs represented different aspects of cell cycle: HIST1H4C is a replication-dependent histone exclusively transcribed during the S-phase of the cell cycle [40,41], UBE2C is required for destruction of mitotic cyclins and cell cycle progression [42], and E2F4 is a transcription factor and exerted a range of functions, mainly in cell cycle, DNA repair, ubiquitination and stress response pathways [43]. In light of the multitude of regulatory functions exerted by E2F4, the significant 2.5 fold up-regulation in PCOS CRCs according to the qRT-PCR experiment might have interesting biological implications, which should be explored in future studies.

## Conclusion

The transcriptomic analysis of CRCs from oocytes developing into embryos selected for transfer showed up-regulation of cell cycle pathways and DNA replication pathways in PCOS CRC samples, however, this had no detectable effect on *in vitro* development of the corresponding embryos. Future studies are needed to clarify whether the up-regulated cell cycle pathways in PCOS CRCs have any clinical implications.

## Additional files

Additional file 1:
**Comparison of the corona radiata cell transcriptome between PCOS and controls (6 PCOS arrays vs. 6 control arrays).** Full gene list showing Log fold change and adjusted p-values. Additional file 2:
**Comparison of the corona radiata cell transcriptome between PCOS and controls (6 PCOS arrays vs. 6 control arrays).** Here is the full list of genes contributing to the upregulation of cell cycle pathways in PCOS corona radiata cells. Mean intensity and fold change of each gene is shown.
